# The Gypsum Influence on the Formation of Secondary Phases During Autoclave Leaching of Gold-Bearing Concentrates and the Silver Recovery Using Cyanidation

**DOI:** 10.3390/ma17215245

**Published:** 2024-10-28

**Authors:** Kirill Karimov, Denis Rogozhnikov, Ilia Fomenko, Alexander Zavalyuev, Maksim Tretiak, Oleg Dizer

**Affiliations:** 1 Laboratory of Advanced Technologies in Non-Ferrous and Ferrous Metals Raw Materials Processing, Institute of New Materials and Technologies, Ural Federal University Named After the First President of Russia B.N. Yeltsin (UrFU), 620002 Yekaterinburg, Russia; kirill_karimov07@mail.ru (K.K.); darogozhnikov@yandex.ru (D.R.); oleg.dizer@urfu.ru (O.D.); 2 R&D Centre for Hydrometallurgy LLC, 196247 Saint Petersburg, Russia; fomenko-i@gidrometall.ru; 3 Pokrovskiy POX Hub, Pokrovskiy Rudnik JSC, 675000 Blagoveshchensk, Russia; zavaluev-a@pokrmine.ru

**Keywords:** autoclave leaching, gypsum, sulfuric acid, lime boiling, sulfide, silver, gold, cyanidation

## Abstract

Autoclave leaching of sulfide concentrates may produce various ferric secondary phases, depending on the arsenic content and temperature. Silver is converted to argentojarosite, from which it is not recoverable by standard cyanidation methods. To increase silver recovery, it is necessary to reduce the argentojarosite formation during autoclave leaching. This study was devoted to the influence of gypsum on the formation of secondary phases of ferric arsenate and the subsequent recovery of gold and silver by cyanidation. The addition of gypsum at a consumption of 0.1 g/g(concentrate) helped to increase silver extraction from 13.4 to 98% at cyanidation. Gold recovery was 99%. An increase in gypsum consumption contributed to the ferric arsenate sulfate formation with an increased sulfate sulfur content, and a decrease in the As/S_(sulfate)_ molar ratio in the cake from 3.7 to 0.88 contributed to an increase in silver extraction at cyanidation of up to 98%. Basic ferric sulfate is not formed in this case, since according to EDS mapping, the distribution of arsenic and sulfur over ferric-containing particles is uniform. According to TCLP, stable, sparingly soluble ferric arsenate phases are formed and the cake obtained after cyanidation is stable and suitable for disposal, since the final arsenic concentration in the solution was 0.45 mg/dm^3^.

## 1. Introduction

Among the various types of ores containing noble metals, those in which gold and silver are in close association with sulfide minerals occupy a special place. These metals are not extracted by cyanidation even after ultrafine grinding of such an ore. According to Ref. [1], the share of refractory ores is more than 30% of the total reserves of noble metals in the world.

This is due to the extremely fine dispersion of gold and silver in sulfide minerals and the presence of noble metals in the crystal lattice of sulfides [2,3,4,5,6,7].

The most reliable and universal methods in the world are those based on the use of pressure oxidation and neutral leaching for the quantitative oxidation of gold- and silver-containing sulfides (pyrite, arsenopyrite, and chalcopyrite) and subsequent cyanidation of the resulting cakes [8,9,10,11,12,13,14,15,16,17,18]. Along with a high gold content, some sulfide concentrates contain significant amounts of silver as well. The extraction of silver from pressure oxidation cakes by cyanidation is difficult due to the formation of argentojarosite by reaction (1) [19,20]:

Ag_2_S + 24.5 O_2_ + FeS_2_ + 15H_2_O = 2AgFe_3_(SO_4_)_2_(OH)_6_ + 18H^+^ + 9SO_4_^2−^ (1)

Argentojarosite is inert towards cyanide ion; therefore, silver extraction during cyanidation of the pressure oxidation residue usually does not exceed 4–20%. To increase silver extraction, it was proposed [21] that we could treat pressure oxidation residues with a lime temperature of 90 °C. The disadvantage of this method is the high consumption of lime (up to 100 kg per ton). Another method [22,23] is that pressure oxidation is carried out with the addition of limestone or lime. Binding of sulfate ions to gypsum inhibits the formation of argentojarosite. However, this method also requires a large consumption of lime and promotes the formation of various deposits, reducing the efficiency of the equipment and affecting its heat balance. There is a known method of steaming pressure oxidation cakes with lime (1.5 times excess wrt stoichiometry, 370 K, L:S = 5:1, duration 5–10 h); however, upon reaching pH = 10, argentojarosite is destroyed according to the following reaction:

2AgFe_3_(SO_4_)_2_(OH)_6_ + 4Ca(OH)_2_ + H_2_O = Ag_2_O + FeOOH + CaSO_4_ × H_2_O (2)

In sulfide concentrates processed in pressure oxidation plants, a significant amount of gold and silver is associated with arsenopyrite, where they are in the form of nano-inclusions of native metal, or in the form of single atoms. A gold atom may take the place of another atom in the structure of arsenopyrite, e.g., replace an atom of iron, arsenic, or sulfur. In another case, gold atoms may be embedded in the voids of the crystal lattice of the mineral, and also occupy “defects” therein (various types of vacancies and dislocations). In the process of pressure oxidation oxidative leaching of arsenopyrite, ferric arsenates are formed: FeAsO_4_·2H_2_O, FeAsO_4_·0.68–0.77H_2_O, and Fe(AsO_4_)_x_(SO_4_)_y_(OH)_z_·wH_2_O, where 0.36 ≤ x ≤ 0.69, 0.19 ≤ y ≤ 0.5, 0.55 ≤ z ≤ 0.8, and 0.2 ≤ w ≤ 0.45 [24,25,26,27,28]. During the conditioning of such cakes, which is necessary to increase gold recovery during subsequent cyanidation, solutions containing Fe (III) and As (V) are formed. They are neutralized with lime to obtain ferric arsenates. Rapid neutralization of solutions with lime is one of the simplest methods for the precipitation of As (III) and As (V) as arsenites and arsenates of iron and calcium at pH = 3–4 [29,30]. The process of lime precipitation is a relatively economical way of immobilizing arsenic; however, the precipitates thus obtained have poor long-term stability [31,32]. Co-precipitation of Fe (III) and As (V) under these conditions occurs in the form of ferrihydride, comprising amorphous ferric arsenates of poorly crystallized scorodite. During long-term storage and exposure to the environment, these compounds are converted to alpha-goethite (α-FeOOH), which leads to the transition of arsenic to the liquid phase [33,34,35,36].

According to Ref. [37], at temperatures below 100 °C, precipitation occurs with the formation of an amorphous solid phase, which consists of gel-like ferric hydroxide containing adsorbed arsenate ions. With an increasing temperature, the crystalline structure of the precipitate improves, and adsorbed arsenate ions, interacting with iron (III), form the scorodite structure. This interaction occurs at temperatures of 150–200 °C with a Fe:As ratio of 1.5 and higher. Ferric arsenates formed under pressure oxidation conditions are more stable in the long term, have a crystalline structure, and are less soluble compared to precipitates obtained under atmospheric conditions.

As a result of hydrothermal interaction, FeAsO_4_ × 2H_2_O, Fe_3_(AsO_4_)_2_SO_4_OH, FeSO_4_OH, and Fe_2_(HAsO_4_)_3_ × xH_2_O can be formed at temperatures of 150 °C and above. The precipitate obtained after 5 min of precipitation at 190 °C already contains crystalline scorodite. It has been established that acidity greatly affects the degree of arsenic precipitation: as it increases, arsenic extraction from the solution decreases (e.g., at 190 °C and 20 g/dm^3^ of free acid, 80% of arsenic is precipitated from the solution).

According to the literature analysis, autoclave leaching of sulfide concentrates may produce various ferric secondary phases, depending on the arsenic content and temperature. Silver is converted to argentojarosite, from which it is not recoverable by standard cyanidation methods. To increase silver recovery, it is necessary to reduce the formation of argentojarosite during autoclave leaching or destroy it afterwards. Ferric arsenates formed by autoclave leaching are more stable in the long term, as they have a crystal structure, and are less soluble compared to precipitates obtained under atmospheric conditions. Application of lime boiling allows us to decompose argentojarosite and to increase silver recovery but favors the decomposition of ferric arsenates and worsens the stability of the obtained cakes. The purpose of this work was to study the effect of a gypsum additive during pressure oxidation leaching of sulfide concentrate containing arsenopyrite on the extraction of gold and silver and the formation of secondary ferric- and arsenic-containing phases.

## 2. Materials and Methods

### 2.1. Analysis and Method

Laboratory experiments on pressure leaching were carried out using a 0.6 dm^3^ autoclave reactor (Parr Instrument, Moline, IL, USA), with openings for sampling. The reactor was thermostated. The materials were stirred using an overhead mixer at 800 rpm, which ensured a uniform density of the pulp. A portion of the raw material weighing 100 g was added to a prepared solution; L:S in all experiments was 5 to 1. The reactor was heated to the required temperature, and then oxygen was supplied, marking the start of the experiment. At the predetermined time intervals, a portion of the leaching pulp was taken and filtered in a Buchner funnel; the solutions were sent for ICP-MS analysis. At the end of the experiment, the leaching cake was washed with distilled water, dried at 100 °C to a constant weight, weighed, and sent for analysis. All the experiments were performed twice, and the mean values are presented here.

Chemical analysis of the starting minerals and the resulting solid dissolution products was carried out using an ARL Advant’X 4200 wavelength dispersive spectrometer (Thermo Fisher Scientific Inc., Waltham, MA, USA). Phase analysis was performed on an XRD 7000 Maxima diffractometer (Shimadzu Corp., Tokyo, Japan).

Particle size analysis was carried out by laser diffractometry using an Analysette 22 Nanotec Plus instrument (FRITSCH GmbH, Idar-Oberstein, Germany).

Chemical analysis of the resulting solutions was carried out by inductively coupled plasma mass spectrometry (ICP-MS) on an Elan 9000 instrument (PerkinElmer Inc., Waltham, MA, USA).

Scanning electron microscopy (SEM) was performed using a JSM-6390LV microscope (JEOL Ltd., Tokyo, Japan) equipped with a module for energy-dispersive X-ray spectroscopy analysis (EDX).

### 2.2. Materials and Reagents

**Figure 1:**
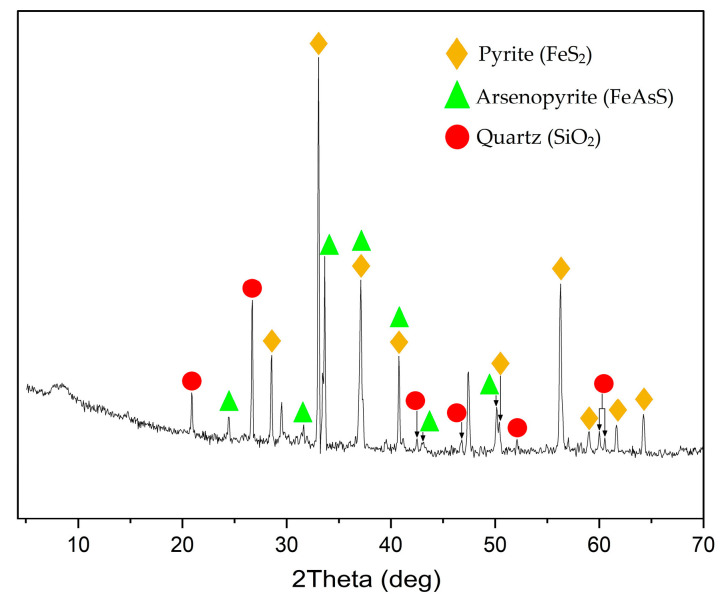
X-ray diffraction pattern of the starting sulfide gold- and silver-containing concentrate.

The main raw material used was a sulfide gold- and silver-containing concentrate of the following composition: Fe–30.7%; As–10.8%; S_total_–32.3%; S_sulfide_–31.9%; Si–3.7%; Au–25.8 g/t; Ag–52.05 g/t. Figure 1 presents its X-ray pattern. According to the data presented, the main minerals in the concentrate are pyrite and arsenopyrite.

**Figure 2:**
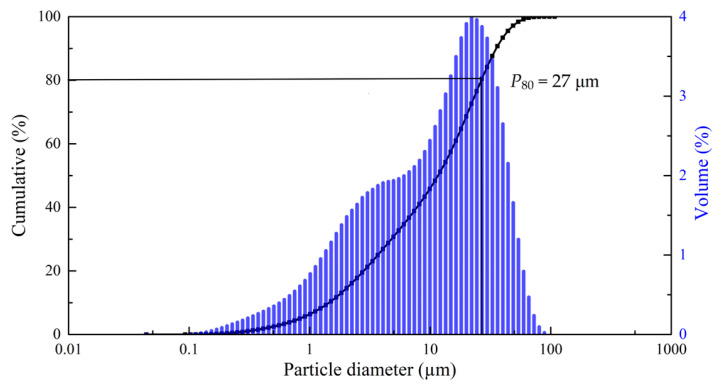
Granulometric composition of the starting concentrate.

The concentrate was sieved on laboratory sieves and analyzed. Experiments were carried out with a fraction of 90% particle size class 20–40 μm, the granulometric composition is presented in Figure 2. All other reagents used were of analytical grade.

### 2.3. Apparatuses

POX experiments were performed in a 1.2 dm^3^ titanium autoclave (Premex AG CH-2543, Lengnau, Switzerland), with the ability to supply and regulate oxygen flow using a flow meter (Bronkhorst EL-FLOW Prestige and Bronkhorst EL-PRESS Metal-Sealed pressure regulators (Bronkhorst High-Tech B.V., Ruurlo, The Netherlands)), and with temperature control. Mixing was carried out using a top-drive mixer to ensure slurry homogeneity.

### 2.4. Stability Evaluation

The stability of arsenic (V) precipitation products was assessed by the Toxicity Characteristic Leaching Procedure (TCLP). To do this, 1 g of a solid sample was added to 20 mL of a buffer solution mixed with acetic acid and sodium acetate (pH~4.9), and then shaken using a shaker at 22 °C for 18 h. The stability of the products was assessed by comparing the resulting arsenic concentration after leaching with the hazardous solid waste standard, which is 5 mg/L for arsenic [30,38].

### 2.5. Cyanidation of Pressure Oxidation Cakes

Pressure oxidation leaching cakes in experiments with gypsum additives were cyanidated without conditioning. Cyanidation was carried out for 24 h in a glass laboratory beaker at room temperature and with mechanical stirring of the slurry. A sample of the material was mixed with distilled water to a solid content of 20% (wt.), and the pH of the slurry was adjusted to 10.5–11.0 using lime milk. After stabilizing this pH, potassium cyanide was added to obtain a concentration of 2 g/L in the solution. If necessary, a concentrated KCN solution was added to the slurry, maintaining the given concentration. Upon completion of the cyanidation process, the slurry was filtered and the cake was washed. The wash water volume was equal to three times the volume of the liquid phase at the cyanidation stage. Next, the cake was dried and the residual content of gold and silver (cyanidation tailings) was analyzed.

## 3. Results and Discussion

### 3.1. Influence of Gypsum Addition on the Behavior of Iron and Arsenic During Pressure Oxidation Leaching, and Silver Recovery During Subsequent Cyanidation of the Residue

A method is known of introducing lime and limestone with pressure oxidation leaching slurry. Binding of sulfate ions to gypsum inhibits the formation of argentojarosite. However, this method also requires a large consumption of lime, with the molar ratio S^2−^:CO_3_ =1:1. According to the data obtained, a positive effect on silver recovery at the stage of cyanidation of pressure oxidation leaching cakes is achieved even with a final acid concentration of 28 g/dm^3^ when all the limestone turns into gypsum [22].

Therefore, we studied the influence of the initial acid concentration, temperature, and gypsum consumption on the behavior of iron, arsenic, and sulfur during pressure oxidation leaching and the subsequent silver recovery during cyanidation. The oxidation degree of concentrate sulfides in all experiments was limited to 95–97%, controlling oxygen consumption, according to the industrial practice [19,20,21,22].

**Figure 3:**
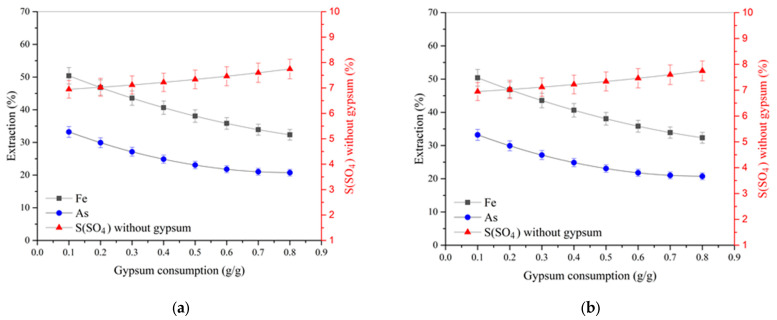

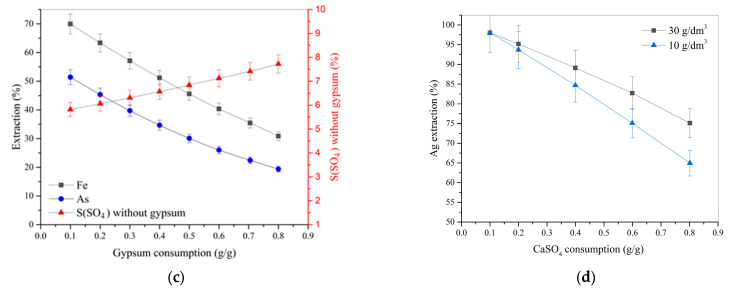
Dependences of the extraction of iron and arsenic into solution and the content of sulfate sulfur not associated with gypsum on gypsum consumption at 225 °C and an acid concentration of (**a**) 10 g/dm^3^, (**b**) 20 g/dm^3^, or (**c**) 30 g/dm^3^. (**d**) Silver recovery as a function of gypsum consumption.

Figure 3 presents the dependence of the extraction of arsenic and iron into solution and the content of sulfate sulfur in the residue not associated with gypsum on gypsum consumption at 225 °C and various initial acid concentrations.

**Figure 4:**
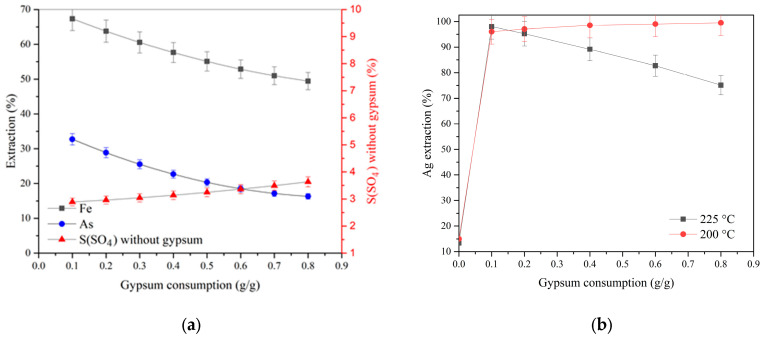
(**a**) Dependence of the extraction of iron and arsenic into solution and the content of sulfate sulfur not associated with gypsum at 200 °C and an acid concentration of 20 g/dm^3^ on gypsum consumption; (**b**) silver extraction vs. gypsum consumption.

According to the data obtained, the extraction degree of arsenic and iron decreases with increasing gypsum consumption, and the slope of the curves is similar at various acid concentrations, which indicates their interaction to form joint compounds—iron arsenates. With an increase in the initial concentration of sulfuric acid from 10 to 30 g/dm^3^, the dependence of the extraction of arsenic and iron on gypsum consumption rises. The content of sulfate sulfur, minus the gypsum added, increases with gypsum consumption, and with an increase in the initial concentration of sulfuric acid from 10 to 30 g/dm^3^, this dependence becomes more pronounced. As can be seen, the content of sulfate sulfur decreases with an increasing acid concentration at low gypsum consumption from 0.1 to 0.5 g/g; its further increase neutralizes the effect of acidity and contributes to obtaining a residue containing 6.6–7.4% sulfate sulfur. Similar things are observed in the process of pressure oxidation leaching at a temperature of 200 °C and an initial acid concentration of 20 g/dm_3_ (Figure 4). With an increase in gypsum consumption from 0.1 up to 0.8 g/g, the content of sulfate sulfur not associated with gypsum increases less noticeably, from 2.9 to 3.6%, while, in contrast to pressure oxidation leaching at 225 °C, silver extraction increases.

The results obtained are consistent with the data of previous research, where it was shown that with an increase in the initial concentration of sulfuric acid, the amount of basic ferric sulfate in the cake (Fe(SO_4_)OH BFS) decreases and the content of ferric arsenate sulfate increases (BFAS (Fe_x_[(SO_4_),(AsO_4_)]_∑1_(OH)_y_·nH_2_O)) [24,25,26,27,39].

Silver recovery increases with an increasing initial sulfuric acid concentration and decreases at 225 °C and gypsum consumption above 0.1 g/g (Figure 3d and Figure 4b). Gypsum introduction occurs when pressure oxidation leaching contributes to a sharp increase in the sulfate sulfur content in the cake from 2.4 to 5.8% at 225 °C and from 1.3 to 3.0% at 200 °C, respectively. In this case, the addition of gypsum at 0.1 g/g of concentrate leads to an increase in silver extraction from 13.4 to 95–98% at the cyanidation stage, but a further increase in consumption above 0.1 g/g promotes a reduction in silver transition to solution at 225 °C and has a positive effect at 200 °C. Gold recovery was 99%.

**Figure 5:**
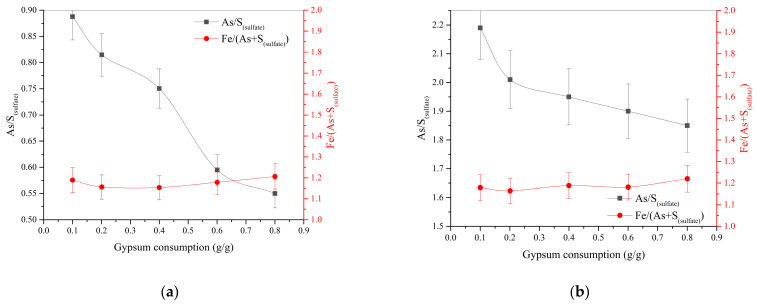
Influence of gypsum consumption on the molar ratio of arsenic to sulfate sulfur not associated with gypsum As/S_(sulfate)_ and the molar ratio of iron to the sum of arsenic and sulfate sulfur Fe/(As + S_(sulfate)_): (**a**) t = 225 °C, H_2_SO_4(initial)_ = 30 g/dm^3^; (**b**) t = 200 °C, H_2_SO_4(initial)_ = 20 g/dm^3^.

Figure 5 shows the effect of gypsum consumption on the molar ratio of arsenic to sulfate sulfur not associated with gypsum As/S_(sulfate)_ and the molar ratio of iron to the sum of arsenic and sulfate sulfur Fe/(As + S_(sulfate)_) in the cake.

An increase in gypsum consumption from 0.1 up to 0.8 g/g during pressure oxidation leaching at 225 °C helps to reduce silver extraction from 98 to 75.1% and the molar ratio of arsenic to sulfate sulfur not associated with gypsum As/S_(sulfate)_ in the cake from 0.88 down to 0.55. At 200 °C, an increase in gypsum consumption affects the As/S_(sulfate)_ ratio in the cake to a lesser extent and helps to increase silver extraction from 96 up to 99.5%, while the Fe/(As + S_(sulfate)_) molar ratio remains almost unchanged and is 1.15–1.21 over the entire range of temperatures, acid concentrations, and gypsum consumption levels. These data show that with an increase in gypsum consumption in cakes, the content of sulfur-associated iron compounds increases, and with an increase in temperature from 200 up to 225 °C, the degree of precipitation of these compounds increases as well. Gold recovery in all experiments was 97–98%.

### 3.2. Analysis of the Resulting Precipitation

**Figure 6:**
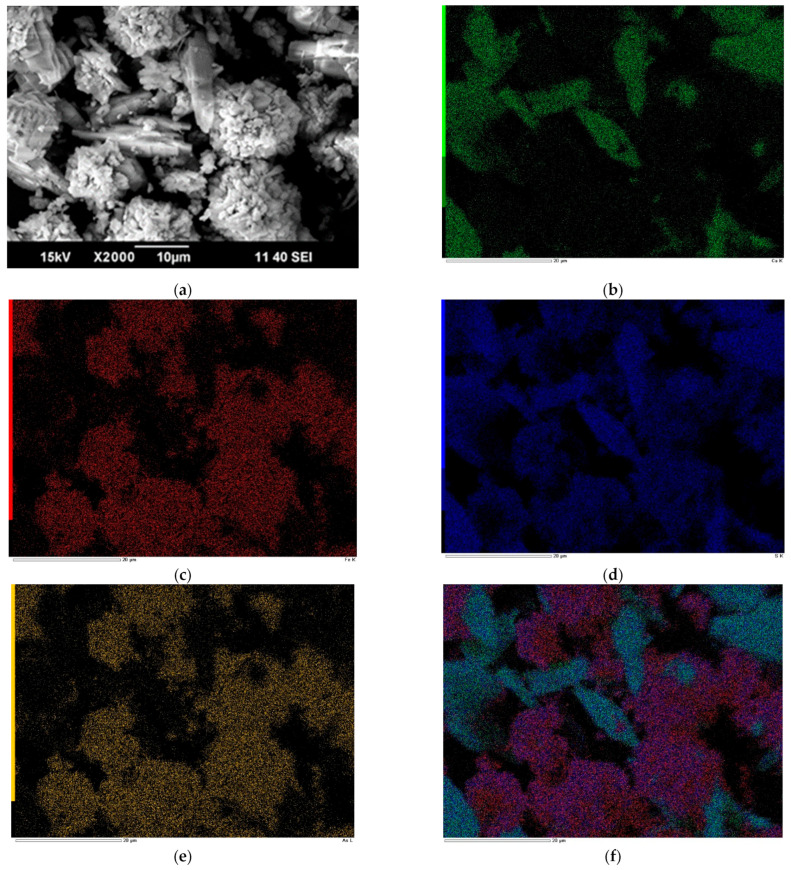
Microphotographs of the precipitate obtained at 225 °C: (**a**) 0.4 g/g gypsum consumption and EDS mapping for (**b**) calcium, (**c**) iron, (**d**) sulfur, (**e**) arsenic, and (**f**) the total.

Figure 6 presents microphotographs of the resulting precipitate at 225 °C, with a gypsum consumption of 0.4 g/g, and its EDS mapping. The resulting cake contains, %: 9.1 As; 20.1 Fe; 8.6 Ca; 12.4 S_(sulfate)_ (As/S_(sulfate)_ = 0.7).

According to Figure 6, the precipitate is represented by needle-shaped particles with a smooth surface and spherical particles with a rough surface. There is a heterogeneous distribution of iron with arsenic and calcium with sulfur (Figure 6), which is caused by the presence of anhydrite (CaSO_4_) and ferric arsenates (Figure 6f).

The EDS mapping results show that arsenic is distributed with iron in one area and is almost absent in the calcium area (Figure 6b,c,e). In the microimages obtained, one can notice the presence of two minerals that may contain Ca, S, Fe, and As, the first being gypsum, and the second being sulfur-containing ferric arsenate. This confirms the formation of ferric arsenates other than scorodite. The distribution of sulfur and arsenic over iron-containing particles is uniform, which indicates the absence of basic ferric sulfate particles in the precipitate.

According to previous research, the peaks on the diffraction patterns of basic ferric sulfate (Fe(SO_4_)(OH)) and basic ferric arsenate sulfate (Fe_x_[(SO_4_),(AsO_4_)]_∑1_(OH)_y_·nH_2_O) have minor differences [24,25,26,27,39].

**Figure 7:**
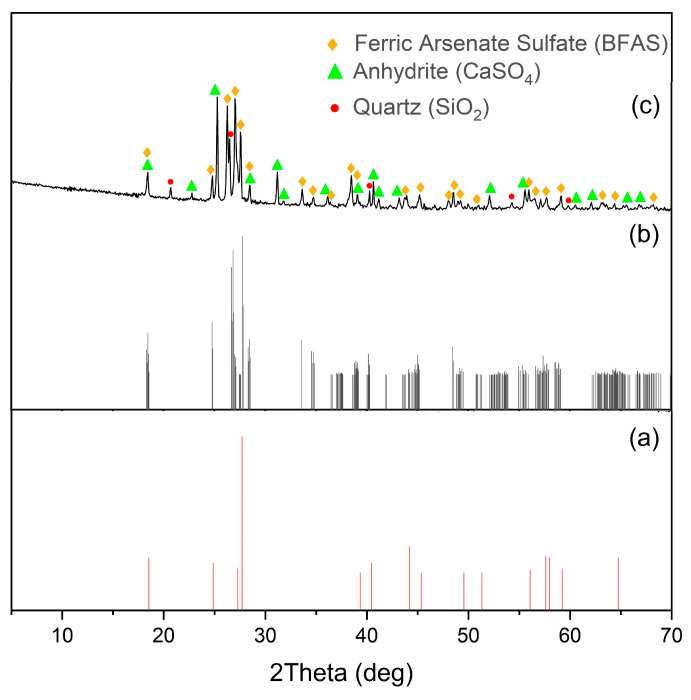
Diffraction patterns of the cakes after pressure oxidation leaching: (**a**) BFS peaks [24,25,26,27,39], (**b**) BFAS peaks (match! Record 96-155-6971), (**c**) t = 225 °C, CaSO_4_ = 0.4 g/g.

Figure 7 shows the diffraction pattern of the cake after pressure oxidation leaching.

As shown in Figure 7, BFAS is present in the precipitate, since its characteristic peaks with angles 2θ = 28.2 and 34.5 were detected. The peaks of BFS coincide with those of BFAS, making it difficult to detect by X-ray diffraction [24,25,26,27,39]. Gypsum recrystallizes into its anhydrite (CaSO_4_).

According to the data obtained, the addition of gypsum contributes to a change in the phase composition of the cake after pressure oxidation leaching. An increase in gypsum consumption promotes the formation of BFAS with an increased content of sulfate sulfur, and a decrease in the As/S_(sulfate)_ molar ratio in the cake from 3.7 to 0.88 contributes to an increase in silver extraction from 13.4 to 98%, while its further decrease from 0.88 to 0.58 leads to a gradual decrease in silver transfer into solution from 98 to 75.1% at the cyanidation stage. This is possibly due to the formation of more crystalline BFAS with an increased sulfate sulfur content with increasing gypsum consumption. The positive effect of acidity on silver recovery and the decrease in sulfate sulfur content minus gypsum could also be explained by a decrease in the formation of BFAS with an increased content of sulfate sulfur, since according to EDS mapping, the distribution of arsenic and sulfur over iron-containing particles is uniform [24,25,26,27].

### 3.3. Study of the Solubility and Toxicity of the Resulting Ferric Arsenate Precipitates

According to Ref. [28], the “Fe(III) oxyhydride–ferrihydrite” phase can sorb AsO_4_^3−^ ions. Sorption occurs through the following reactions:

Fe^3+^ + (3 + x)H_2_O ⇄ FeO(OH)(H_2_O)_1+x_ + 3H^+^ (3)

FeO(OH)(H_2_O)_1+x_ + AsO_4_^3−^ ⇄ AsO_4_^3−^ × FeO(OH)(H_2_O)_1+x_ (4)

When these precipitates are stored for a long time in atmospheric conditions, reactions (3) and (4) may proceed in the opposite direction, thereby creating a danger of environmental contamination with toxic, water-soluble arsenic compounds. Therefore, the solubility of arsenic from precipitates is one of the most important indicators for organizing their long-term storage.

The toxicity of the precipitate was tested on pressure oxidation leaching cake after cyanidation, obtained at a temperature of 225 °C, oxygen pressure of 0.5 MPa, gypsum consumption of 0.4 g/g, and the following composition, %: 9.1 As; 20.0 Fe; 15 S; 8.6 Ca. The cake was washed in distilled water and kept for 24 h at temperatures of 24–26 °C with periodic stirring. After washing and measuring the arsenic concentration in the liquid phase, 1 g of the solid sample was added to 20 cm^3^ of a buffer solution mixed with acetic acid and sodium acetate (pH~4.9) and stirred for 18 h according to the TCLP method [30,39].

The TCLP analysis shows that the cake produced from pressure oxidation leaching and cyanidation is stable and safe for disposal. The final concentration of arsenic in the solution was 0.45 mg/dm³, which is well below the allowable threshold of 5 mg/dm³ for this method.

## 4. Conclusions

In this work, studies were carried out on the extraction of silver and gold from cakes of pressure oxidation leaching of sulfide concentrate containing pyrite and arsenopyrite. The addition of gypsum at a consumption of 0.1 g/g of concentrate helps to increase silver extraction from 13.4 to 95–98% at the cyanidation stage, with no conditioning operation. Gold recovery was 99%.

An increase in gypsum consumption contributes to the formation of BFAS with an increased content of sulfate sulfur, and a decrease in the As/S_(sulfate)_ molar ratio in the cake from 3.7 down to 0.88 contributes to an increase in silver extraction at the cyanidation stage up to 98%, and its further decrease from 0.88 down to 0.58 leads to a gradual reducing silver transfer into solution from 98 down to 75.1% at the cyanidation stage. This is possibly due to the formation of more crystalline BFAS with an increased sulfate sulfur content with increasing gypsum consumption. The positive effect of acidity on silver recovery and the reduction of sulfate sulfur minus gypsum may also be explained by the reduction in the formation of BFAS with an increased sulfate sulfur content. Basic ferric sulfate (BFS) is not formed in this case, since according to EDS mapping, the distribution of arsenic and sulfur over iron-containing particles is uniform.

According to TCLP analysis, the cake obtained after pressure oxidation leaching with the addition of gypsum and cyanidation is stable and suitable for disposal, since the final concentration of arsenic in the solution was 0.45 mg/dm^3^.

## Data Availability

The original contributions presented in the study are included in the article, further inquiries can be directed to the corresponding author.
